# A New Strategy for Treating Renal Fibrosis Based on a Drug‐Food Homogeneous Formula of Traditional Chinese Medicine

**DOI:** 10.1002/fsn3.71186

**Published:** 2025-11-14

**Authors:** Gao Zitian, Tang Yuyan, Huang Luyan, Xu Jiahui, Huang Lusheng, Sun Weiqian, He Haidong, Chen Yu

**Affiliations:** ^1^ Key Laboratory for Advanced Materials and Joint International Research Laboratory of Precision Chemistry and Molecular Engineering, Feringa Nobel Prize Scientist Joint Research Center, School of Chemical and Molecular Engineering East China University of Science and Technology Shanghai China; ^2^ Department of Nephrology, Minhang Hospital Fudan University Shanghai China; ^3^ Fudan Zhangjiang Institute Shanghai China

**Keywords:** colonic dialysis, drug‐food homogeneous formula, intestinal barrier, PI3K/AKT pathway, renal fibrosis

## Abstract

Renal fibrosis is a common pathological feature of chronic kidney disease (CKD) as it progresses to the end stage. Currently, about 10% of adults worldwide are affected by this disease. In recent years, significant progress has been made in the field of renal fibrosis treatment, with both traditional drugs and innovative therapies showing new hope. In this study, we propose a novel therapeutic strategy for renal fibrosis based on the traditional Chinese medicine concept of “medicinal and edible homology”. This involves the use of a compound formulation comprising red ginseng, dandelion, and oyster shell, and evaluating its therapeutic effect on renal fibrosis through the colon administration route. Through integrated network pharmacology and molecular docking analyses, we identified AKT1, JUN, TNF, HSP90AA1, and IL‐6 as core targets, with the PI3K/AKT signaling pathway playing a key role. Colon administration of the formulation significantly restored intestinal barrier function by upregulating tight junction proteins ZO‐1 and occludin, thereby reducing circulating endotoxin (LPS) and systemic inflammatory markers (IL‐6, IL‐1β). Concurrently, the treatment intervened in the renal PI3K/AKT pathway, upregulated expressions of PI3KR1, PKCa, and AKT1/2/3, improved renal function (reduced creatinine and urea nitrogen), and ameliorated fibrosis markers (reduced fibronectin and α‐SMA, increased E‐cadherin). These findings demonstrate that the medicinal‐edible homologous formulation alleviates renal fibrosis via modulation of the gut–kidney axis, combining intestinal barrier repair with suppression of pro‐fibrotic signaling. This study supports the use of colon‐delivered traditional Chinese medicine as a promising therapeutic strategy for renal fibrosis.

## Graphical abstract

Renal fibrosis is a common pathological feature of chronic kidney disease as it progresses to the end stage. This study explores a novel medicinal‐edible homologous formulation delivered via colon to target the gut–kidney axis. The formulation repaired the intestinal barrier, reduced systemic LPS and inflammation, and subsequently suppressed renal fibrosis by modulating the PI3K/AKT pathway, ultimately improving renal function. Our work demonstrates that the colon‐delivered herbal strategy is a promising therapeutic approach for combating renal fibrosis.

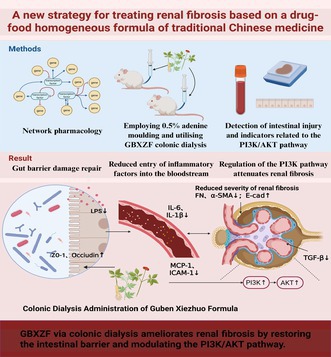

## 1 Introduction

Chronic kidney disease (CKD) is a common progressive disease, affecting more than 10% of the global population (Kovesdy 2022). Epidemiological projections indicate that CKD is expected to emerge as the fifth leading cause of global mortality by 2040 (Foreman et al. 2018; Kovesdy 2022). Renal fibrosis is a pathological feature of CKD; distinct histopathological manifestations characterize this fibrotic process, including glomerulosclerosis, tubulointerstitial fibrosis, and inflammatory cell infiltration. The progression of renal fibrosis contributes significantly to the exacerbation of CKD and the eventual development of end‐stage renal disease (ESRD). As the molecular mechanisms of fibrosis become increasingly elucidated, an expanding array of therapeutic agents for renal fibrosis is emerging. Currently, the treatment of CKD includes symptomatic treatment with renin‐angiotensin‐aldosterone system (RAAS) drugs, sodium‐glucose cotransporter protein 2 (SGLT2) inhibitors, and immunosuppressants to improve proteinuria, hypertension, and hyperglycemia, and to slow down the progression of CKD (Lazzaroni et al. 2022; Maringhini and Zoccali 2024). Recognizing the limitations of current symptom‐centered therapies, we have developed an innovative “food as medicine” strategy to alleviate renal fibrosis through a systematic, holistic, integrative approach.

According to traditional Chinese medicine (TCM), the pathology of CKD is primarily attributed to the spleen and kidney, with the main etiology being the deficiency of these organs and the internal production of turbid toxins. A combination of underlying deficiency and superficial excess often characterizes the disease mechanism of CKD. Therefore, activating blood circulation and removing blood stasis, clearing heat and removing toxins, tonifying the spleen and kidney, and supporting the positive and dispelling the evil spirits are the directions of TCM in treating CKD and resisting renal fibrosis (Li, Xu, et al. 2022; Liu, Zhang, Wang, et al. 2022; LLiu, Zhang, Zhao, et al. 2022; Wang, Feng, et al. 2022). Notably, the concept of “homology of medicine and food” offers a unique perspective for CKD management. Many natural substances, such as Chinese yams, goji berries, and poria, are not only nutritional foods but also possess pharmacological properties that can modulate the intestinal microenvironment, alleviate inflammation, and improve oxidative stress—all of which are highly relevant to the pathophysiology of CKD (Lu et al. 2023).

Building upon the concept of “medicinal‐food homology,” our group developed a formula, GBXZF, based on drug‐food homologous ingredients including red ginseng, dandelion, and oysters. This formulation is designed to integrate nutritional support with pharmacological activity. Preliminary studies confirmed that GBXZF colonic dialysis reduces inflammatory cell infiltration and renal fibrosis (RIF) levels in uremic rat models (Li, Xu, et al. 2022), though its therapeutic effects on renal fibrosis remain unclear. Therefore, we systematically investigated GBXZF's molecular mechanisms for improving renal fibrosis through integrated bioinformatics analysis and in vivo validation.

## 2 Materials and Methods

### 2.1 Reagents

LC–MS grade acetonitrile (ACN) and methanol (MeOH) were purchased from Merck (USA). Pure distilled water was acquired from Watsons (Guangzhou, China). Formic acid from CNW was of HPLC grade. The Guben Xiezhuo formula comprises Prepared Aconite, Red Ginseng, Carthamus Flower, Prepared Rhubarb, Dandelion, and Oyster Shell. The above‐mentioned medicinal materials were supplied by the Traditional Chinese Medicine Department of Minhang Hospital, affiliated with Fudan University. Male SD rats, 6–8 weeks old and weighing 180–200 g, were purchased from Shanghai Mai Bo Biotechnology Co. Ltd.

### 2.2 Preparation and Characterization of Guben Xiezhuo Formula

The Guben Xiezhuo formula, its composition, and clinical application plan: Prepared Rhubarb 30 g, Prepared Aconite 10 g, Red Ginseng 10 g, Oyster Shell 50 g, Carthamus Flower 10 g, Dandelion 30 g. The above six medicinal materials were decocted three times, concentrated to 500 mL, and stored in a 4°C refrigerator. The sample was subjected to centrifugation at 12,000 rpm for 5 min. UPLC‐Q‐TOF‐MS characterized the obtained supernatant.

Chromatographic detection was performed using a 1290 UPLC (Ultra‐Performance Liquid Chromatography) system (Agilent, Santa Clara, CA, USA). An Agilent ZORBAX RRHD SB‐Aq column (2.1 × 100 mm,1.8 μm) was used at a flow rate of 0.3 mL min^−1^, and the column temperature was maintained at 30°C. The injection volume was 2 μL, and the detection wavelength ranged from 190 to 400 nm. Solution A was acetonitrile, while solution B was a 0.1% solution of formic acid in water. The gradient of the mobile solution was as follows: 0–3 min, 3% A; 3–7 min, 3%–10% A; 7–15 min,10%–25% A; 15–25 min, 25%–40% A; 25–30 min, 40%–90% A; 30–35 min, 90% A; 35–35.1 min, 90%–3% A; 35.1–38 min,3% A.

Mass spectrometry detection was carried out using a Q‐TOF 6545 LC/MS (Liquid Chromatography/Mass Spectrometry) system (Agilent, Santa Clara, CA, USA). The operational parameters included a gas temperature of 325°C, a gas flow rate of 8 L min^−1^, a nebulizer pressure of 45 psi, a capillary voltage of 4000 V, a fragment voltage of 175 V, and a skimmer voltage of 60 V. Full‐scan MS spectra were acquired within the mass‐to‐charge ratio (m/z) range of 50–1700. The targeted MS/MS collision energies were set at 40 eV. A reference solution was infused for continuous calibration, with m/z values of 121.0508 and 922.0097 utilized for positive mode calibration and m/z values of 197.8073 and 1033.9881 for negative mode calibration.

### 2.3 Filtration of Active Ingredients and Target Diseases

The drug's active ingredients were derived from UPLC‐Q‐TOF‐MS analysis and entered into the PubChem database (https://pubchem.nc‐bi.nlm.nih.gov/) to derive the corresponding SMILE formula. The SMILE formulas were introduced into the SIB database (https://www.swisstargetprediction.ch/), and the action target was obtained with the condition limited to Homosapiens. After removing the duplicate entries, potential targets for the Guben Xiezhuo formula were constructed. In the meantime, “chronic kidney disease” was brought into the GeneCards (https://www.genecards.org/) database and the OMIM (https://www.omim.org/) database to research disease‐related targets. All targets were integrated into Excel; duplicate genes were eliminated and merged to be the disease targets. Potential targets of active ingredients were matched to CKD disease targets by microbiome informatics (http://www.bioinformatics.com.cn). The obtained active ingredient‐CKD co‐action targets were therapeutic targets.

### 2.4 Network Pharmacology and Molecular Docking

The active ingredients and targets of the Guben Xiezhuo formula were imported into Cytoscape 3.9.2 software for network topology analysis. The target graph, color, transparency, and size were adjusted to construct a network diagram of “TCM component‐target‐disease” and screen out the core components according to the Degree value (the number of gene connections). In addition, the typical targets were entered into STRING (https://string‐db.org/) to obtain the PPI relationship, with the following parameters set: specifying Homo sapiens as the species condition, applying a confidence score of 0.9, and hiding free‐floating gene nodes, with other parameters unchanged. The data were added into Cytoscape 3.9.2 software to obtain the PPI maps, and the top 10 core targets were filtered based on Degree values. Subsequently, core targets were imported into the DAVID database (http://www.david.niaid.nih.gov) for GO and KEGG pathway enrichment analyses. The KEGG and GO enrichment analyses were visualized by the microbiome informatics (http://www.bioinformatics.com.cn).

Based on the foregoing research findings, the CytoNCA plugin was utilized to select the top five core genes as receptors and the top five core components as ligands for molecular docking. The 3D structure of the ligand can be acquired from the PubChem3D database (https://pubchem.ncbi.nlm.nih.gov/), and the 3D structure of the receptor can be obtained from the UniProt database (https://www.uniprot.org/). The Pymol 2.2.0 software was used to add hydrogen atoms and remove water molecules and any residual ligands. AutoDock 1.5.6 software was finally applied to simulate the docking of key protein receptors with active ingredients, and their binding conformations were visualized using Discovery Studio 2021 Client software.

### 2.5 Rat Model and Treatment

Animal experiments were approved by the Animal Ethics Committee of the Minhang Hospital, Fudan University (Shanghai, China; *No.2023‐MHFY‐26JZS*). Take care of and treat rodents according to the guiding principle established by the Shanghai Public Health Service Policy on the Humane Care and Use of Laboratory Animals. SD rats were kept in plastic cages in the Animal Experiment Centre of Minhang Hospital, Fudan University. They were housed at a relatively suitable temperature and humidity, with free intake of chow and water, and experiments were carried out after 1 week of adaptive feeding.

The modeling methods were referred to as those in the literature with appropriate modifications (Thakur et al. 2018). The healthy male SD rats were randomly divided into four groups: control (control group, standard chow feeding), CKD (CKD model group, 0.5% adenine chow feeding), NSG (CKD model group, 0.5% adenine chow feeding, and saline enema), and GBXZF (Guben Xiezhuo formula group, 0.5% adenine chow feeding, and enema Guben Xiezhuo formula) (*n* = 6 per group). Enemas were administered after 8 weeks of continuous feeding with 0.5% adenine or standard chow. General conditions, including the rats' body weight, hair, and behavior, were dynamically observed and recorded. The enema tube of a homemade enema device was inserted into the rat's anus approximately 10 cm to perform the enema, and the enema was retained for 30 min.

Before the enema procedure, blood was collected from the rats' tails to measure biochemical indicators. At the end of 4 weeks of enemas, blood was obtained from the abdominal aorta under 2.5% tribromoethanol anesthesia for biochemical indicators and ELISA testing. After cervical dislocation, kidney and colon tissues were taken for histopathological assay, immunohistochemistry, and qRT‐PCR.

### 2.6 Biochemical Indicator Tests

The blood obtained in the experiment was centrifuged (3000 rpm, 4°C, 15 min), and the supernatant, which is the serum, was used for the detection of SCr (serum creatinine) and BUN (blood urea nitrogen). Creatinine assay kits, urea nitrogen assay kits, and uric acid assay kits (#C011‐2‐1, #C013‐2‐1, Nanjing Jiancheng Technology Co. Ltd., Nanjing, China) were used for the detection of biochemical indicators, and the specific operational steps followed the instructions provided with the assay kits.

### 2.7 Enzyme‐Linked Immunosorbent Assay (ELISA)

The Elisa kit (#EK301B, #EK306, #EK306, #EK387,389, Multisciences, Hangzhou, China) was utilized to detect four inflammatory factors, IL‐1β, IL‐6, MCP‐1, and ICAM‐1, in rat serum. The procedure was described in the instruction manual.

### 2.8 Histopathological Assay

Kidneys and colons were fixed in 4% paraformaldehyde and then embedded in paraffin. H&E staining (hematoxylin–eosin staining) and Masson's staining were applied to observe the extent of pathological damage, inflammation, and fibrosis. Images were scanned by the NIKON DS‐U3 (Japan) and further processed using Case Viewer 2.4.0 and Image J 1.48 software.

### 2.9 Immunohistochemistry

Paraffin sections were blocked and serum‐occluded after antigen repair with citrate antigen repair buffer (pH 6.0). The primary antibodies from ABclonal (#A25907, #A7248, #A20798, #A25306, #A24601) (Wuhan, China) and the secondary antibodies from SeraCare (#5220–0362, #5220–0336, #5220–0341, #5220–0364) (Milford, USA) and DAKO (#K5007) (Copenhagen, Denmark) were applied to the sections according to the specific markers to be detected, namely FN (fibronectin; 1:200), a‐SMA (alpha‐smooth muscle actin; 1:200), E‐cadherin (1:200), ZO‐1 (zonula occludens‐1; 1:200), and occludin (1:400). Finally, the sections were color‐developed, re‐stained, and closed. Case Viewer 2.4.0 and Image J 1.48 software were used for processing and quantitative analysis of immunohistochemical maps.

### 2.10 Statistical Analysis

All experimental data were presented as the mean ± standard deviation (SD). Statistical significance was determined by SPSS Statistics 23.0 (SPSS, Chicago, IL, USA) using the Student's *t*‐test and one‐way ANOVA. The diagrams were created using GraphPad Prism 8.3.0 (GraphPad Software Inc., CA, USA).

## 3 Results

### 3.1 Visual Analysis of Network Pharmacology

**FIGURE 1:**
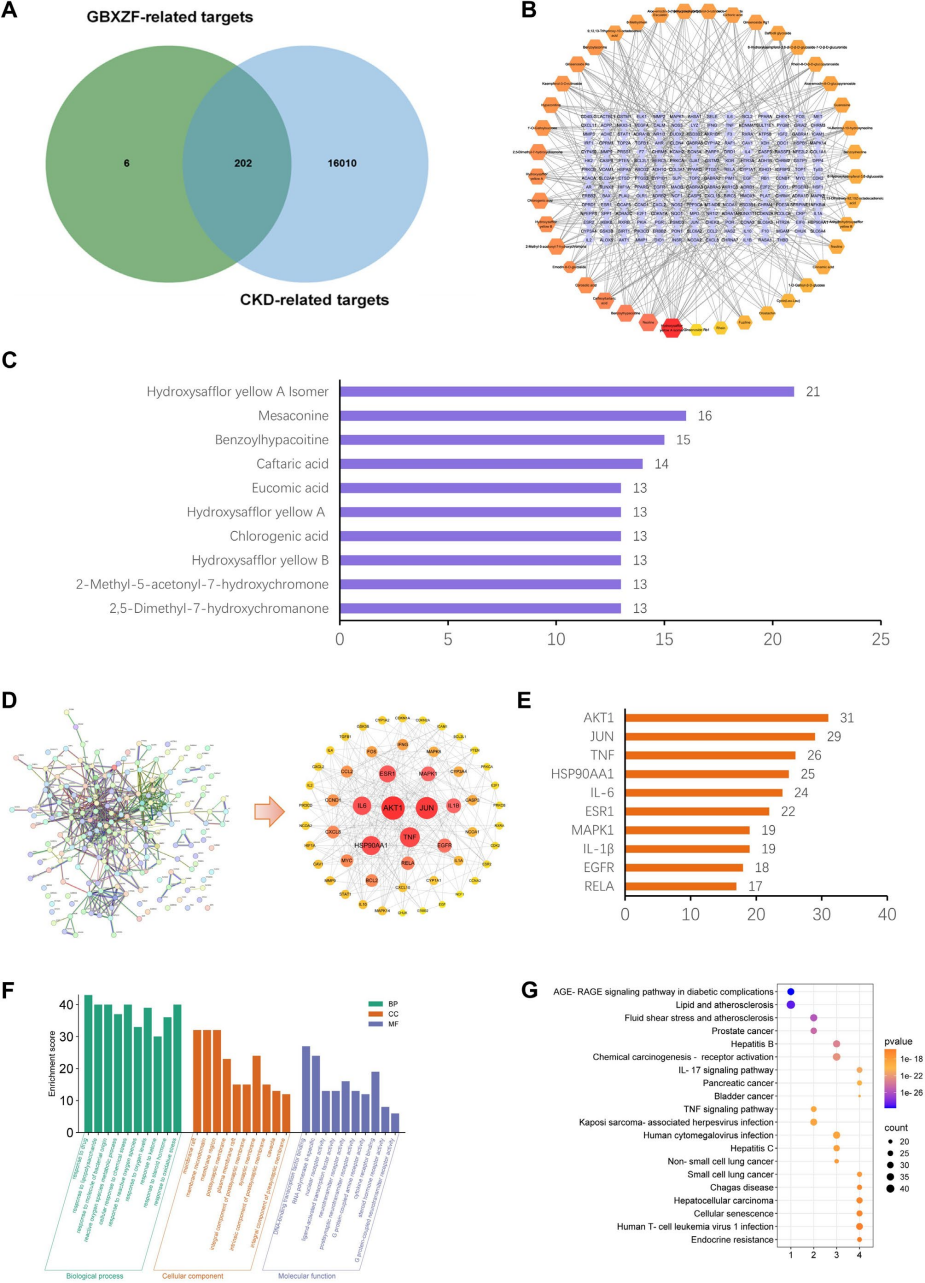
Visualization of network pharmacology. (A) Venn plot showed overlapping targets between the GBXZF active ingredient and CKD. (B) “Active components‐targets‐diseases” network diagram for GBXZF and CKD. (C) Core active ingredients of GBXZF. (D) PPI network map of GBXZF and CKD. (E) The core targets of the joint action of GBXZF and CKD. (F) GO enrichment analysis of the intersection targets. (G) KEGG enrichment analysis of the intersection targets.

To investigate the specific mechanism of action of the GuBen Xiezhuo formula in the treatment of CKD, we carried out a network pharmacological analysis. The mass spectral identification of GBXZF is shown in Figure SS1 and Table S1. The potential targets of the 208 active ingredients of GBXZF were matched with 16,212 CKD disease targets, and 202 active ingredient–CKD co‐targets of GBXZF were obtained (Figure 1A). The active ingredients and potential targets of GBXZF were imported into Cytoscape 1.7. 2 software, and a network diagram of active ingredients–targets–diseases of GBXZF was constructed. The top 10 core components were screened according to the Degree value, which were hydroxy saffron yellow pigment A isomer, neoaconitine, benzoyl hypaconitine, caffeoyl tartaric acid, and erythrocyanic acid, etc. (Figure 1B,C). After that, the co‐interacting targets obtained through the Wayne diagram were imported into the String database to analyze the interactions between GBXZF and CKD intersecting targets, and the PPI network diagram was obtained. The core targets were screened according to the Degree value, and the top 10 targets were rounded up as AKT1, JUN, TNF, HSP90AA1, IL6, etc. (Figure 1D,E). The 202 intersecting targets were imported into the DAVID database, and the top 10 GO entries were filtered by *p* value size for visual analysis. The results show that the biological processes associated with these targets mainly include response to drugs, response to lipopolysaccharide, and response to molecules of bacterial origin; cellular components mainly involve membrane raft, membrane microdomain, and membrane region; molecular functions mainly involve DNA‐binding transcription factor binding, RNA‐polymerase II‐specific–DNA‐binding transcription factor binding, and nuclear receptor activity (Figure 1F). KEGG enrichment analysis revealed 186 pathways, and the targets enriched in KEGG pathways mainly include the AGE‐RAGE signaling pathway in diabetic complications, Lipid, and atherosclerosis, Fluid shear stress and atherosclerosis, IL‐17 signaling pathway, TNF signaling pathway, etc. (Figure 1G).

### 3.2 Molecular Docking Simulation of Active Ingredients and Target Proteins

**FIGURE 2:**
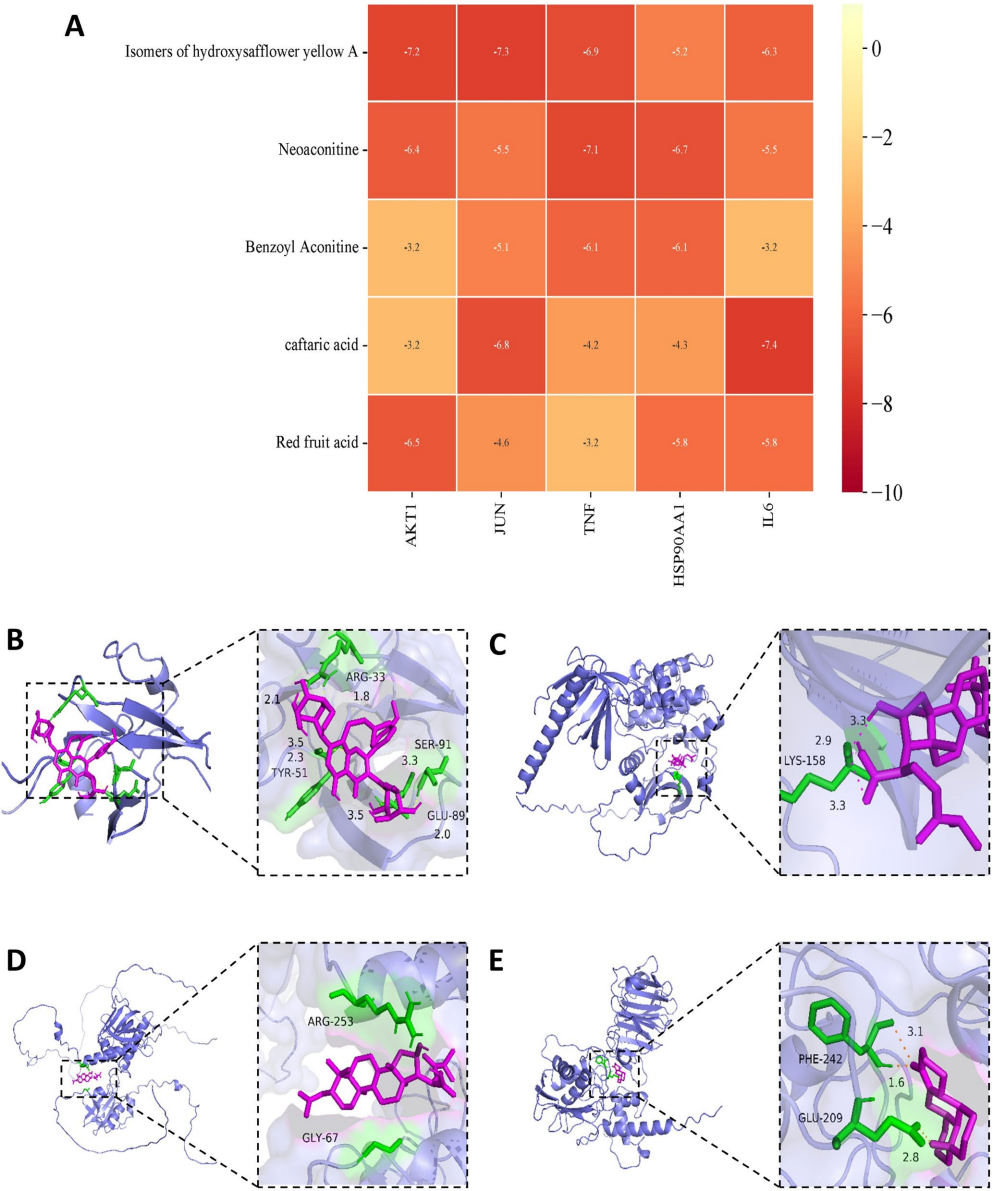
Molecular docking simulation of the active ingredients with core targets. (A) Heat map of molecular docking between the active ingredients of GBXZF and the core targets of CKD. (B–E) Molecular docking of GBXZF active ingredients with core targets: (B) caftaric acid with IL6; (C) hydroxysaffron yellow A isomer with AKT1; (D) hydroxysaffron yellow A isomer with JUN; (E) neoaconitine with TNF.

The binding energies of the docking results of the five active ingredients of the Solid Formula and the five core targets are shown in Figure 2A. In Figure 2A, most active ingredients' binding energies to the core targets were less than −5 kcal·mol−1, indicating a good binding of the receptor to the ligand. The binding energies of hydroxy saffron yellow pigment A isomer, the active ingredient of Guoben Xiezhuo Formula, to the core targets of AKT1 and JUN, were all less than −7 kcal·mol−1. The binding energy of caffeoyl tartaric acid to the core target IL6 and neo aconitine to the core target TNF is also less than −7 kcal·mol−1, which suggests that the active ingredients of the formula can be better combined with the core target of CKD, and the potential biological activity is higher. The molecular docking results of the active ingredients with binding energies less than −7 kcal·mol−1 and the core targets were represented in 3D plots (Figure 2B–2E). Among them, AKT1 and IL‐6 are the core targets and key downstream factors of the PI3K/AKT inflammatory pathway, respectively, which suggests that the mechanism of action of Solid Formula to improve renal fibrosis may be related to the PI3K/AKT pathway.

### 3.3 GBXZF Improves Renal Fibrosis

**FIGURE 3:**
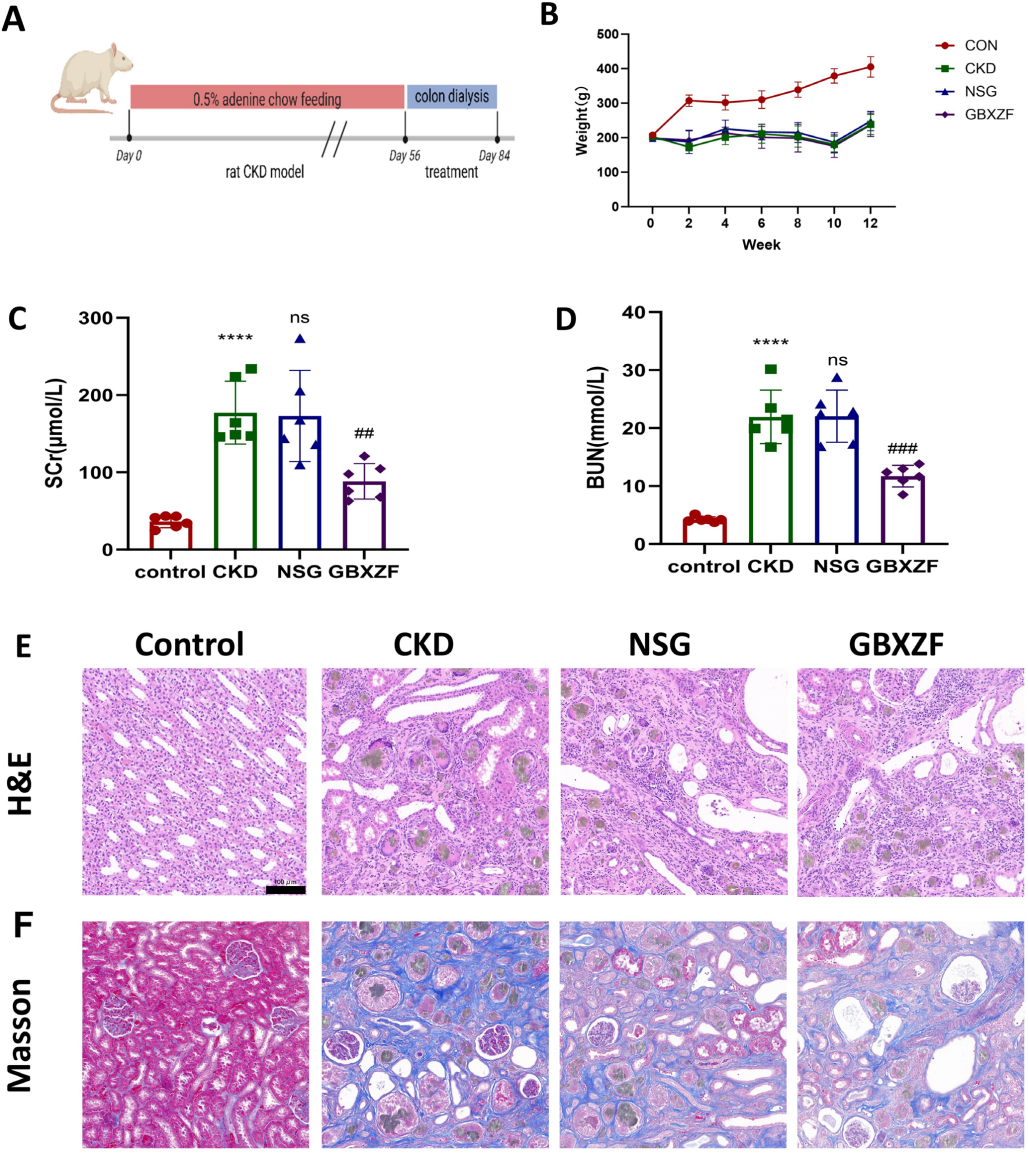
GBXZF alleviated renal fibrosis. (A) The protocol of the animal experiment. (B) The weight alteration trend for all groups. (C, D) Renal function assay conducted by measuring creatinine and blood urea nitrogen. (E, F) Representative renal pathological sections of the H&E and Masson's trichrome staining. Scale bar = 100 μm; magnification, × 20. Data represent mean ± SD (*n* = 6).*p < 0.05, **p < 0.01,***p < 0.001 and ****p < 0.0001.

Combining the results of network pharmacology and molecular docking, animal experiments were conducted to further explore the specific molecular mechanisms by which GBXZF intervenes in renal fibrosis. The design of the animal experiment is shown in Figure 3A. Initially, we established a CKD rat model and treated CKD rats fed with 0.3% adenine with GuBen XieZhuo formula (GBXZF) through colonic dialysis. Compared to the control group, the CKD group rats had sparse and dull fur, were listless, had poor responsiveness, significantly reduced food and water intake, decreased urine volume, dry feces, and significantly lower body weight. The NSG group rats showed no significant difference from the CKD group rats. The GBXZF group rats had substantially better general conditions, such as fur color and mental state, compared to the NSG group rats, but there was no significant difference in body weight between the two groups (Figure 3B). We measured the renal function indicators of the four groups of rats and analyzed the renal pathology of rats using HE staining and Masson staining. Compared to the control group, the levels of Scr and BUN in the CKD group rats were significantly increased, indicating successful modeling with kidney damage. There was no significant change in the NSG group rats compared to the CKD group rats, while the levels of Scr and BUN in the GBXZF group rats were significantly reduced compared to the NSG group, suggesting improved renal function in rats (Figures 3C and 3D). Pathological results showed that the renal tissue structure of the control group was normal, while the CKD and NSG groups had severe renal tissue damage, with a large amount of urate deposition, numerous inflammatory cells, and increased collagen fibers. The degree of renal tissue damage in the GBXZF group was significantly improved compared to the NSG group, with some urate deposition still visible, but a significant reduction in collagen fibers and inflammatory cells (Figure 3E,F).

### 3.4 GBXZF Ameliorates Systemic Inflammatory Response and Renal Fibrosis

**FIGURE 4:**
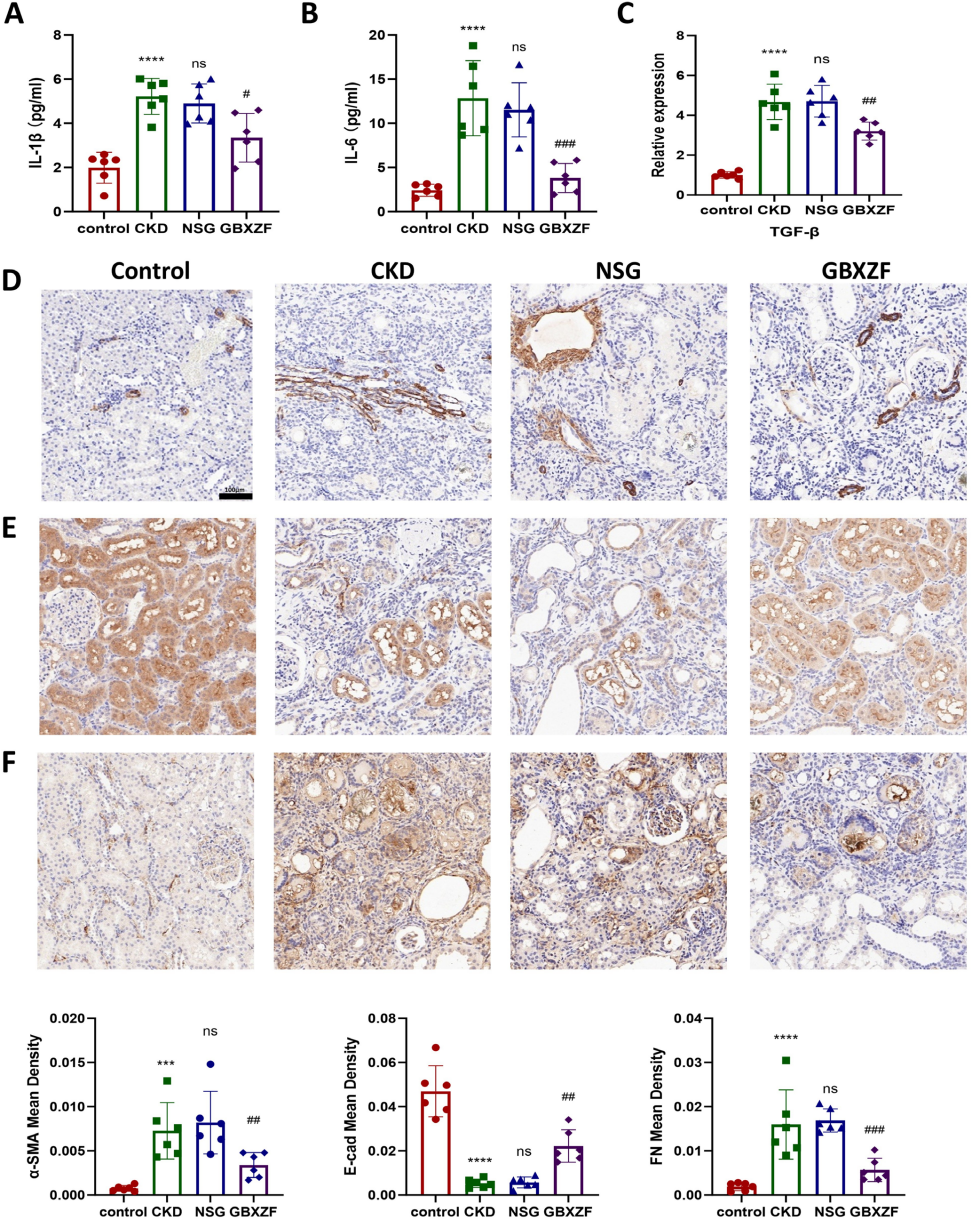
GBXZF ameliorates systemic inflammatory response and renal fibrosis. The serum levels of (A) IL‐1β and (B) IL‐6. Relative expression of the (C) TGF‐β in kidney tissue in the control, CKD, NSG, and GBXZF rats. Immunohistochemistry images and protein mean densities of (D) α‐SMA, (E) E‐cad, and (F) FN in the kidney of the rats from the control, CKD, NSG, and GBXZF rats. Data represent mean ± SD (*n* = 6). *p < 0.05, **p < 0.01,***p < 0.001 and ****p < 0.0001.

To assess the impact of GBXZF on systemic inflammation and key fibrosis markers, we measured serum cytokine levels and renal protein expression. Compared with the control group, serum levels of pro‐inflammatory cytokines IL‐1β and IL‐6 were significantly elevated in the CKD group (Figure 4A,B). This systemic inflammatory response was accompanied by significantly increased renal expression of the fibrosis marker TGF‐β, as well as the extracellular matrix (ECM) components α‐SMA and Fibronectin (FN), and significantly decreased expression of the epithelial marker E‐cadherin (E‐cad) (Figure 4C–F). The NSG group showed no significant changes in serum IL‐1β, IL‐6, or renal TGF‐β, α‐SMA, FN, and E‐cad expression compared to the CKD group. In contrast, GBXZF treatment significantly reduced serum levels of IL‐1β and IL‐6 compared to the NSG group (Figure 4A,B). This reduction in systemic inflammation was associated with significantly decreased renal expression of the fibrosis markers TGF‐β, α‐SMA, and FN, and significantly increased expression of E‐cad compared to the NSG group (Figure 4C–F). These results demonstrate that GBXZF reduces circulating pro‐inflammatory cytokines and attenuates renal fibrosis by decreasing profibrotic markers and restoring epithelial integrity.

### 3.5 GBXZF Repairs Intestinal Barrier Damage

**FIGURE 5:**
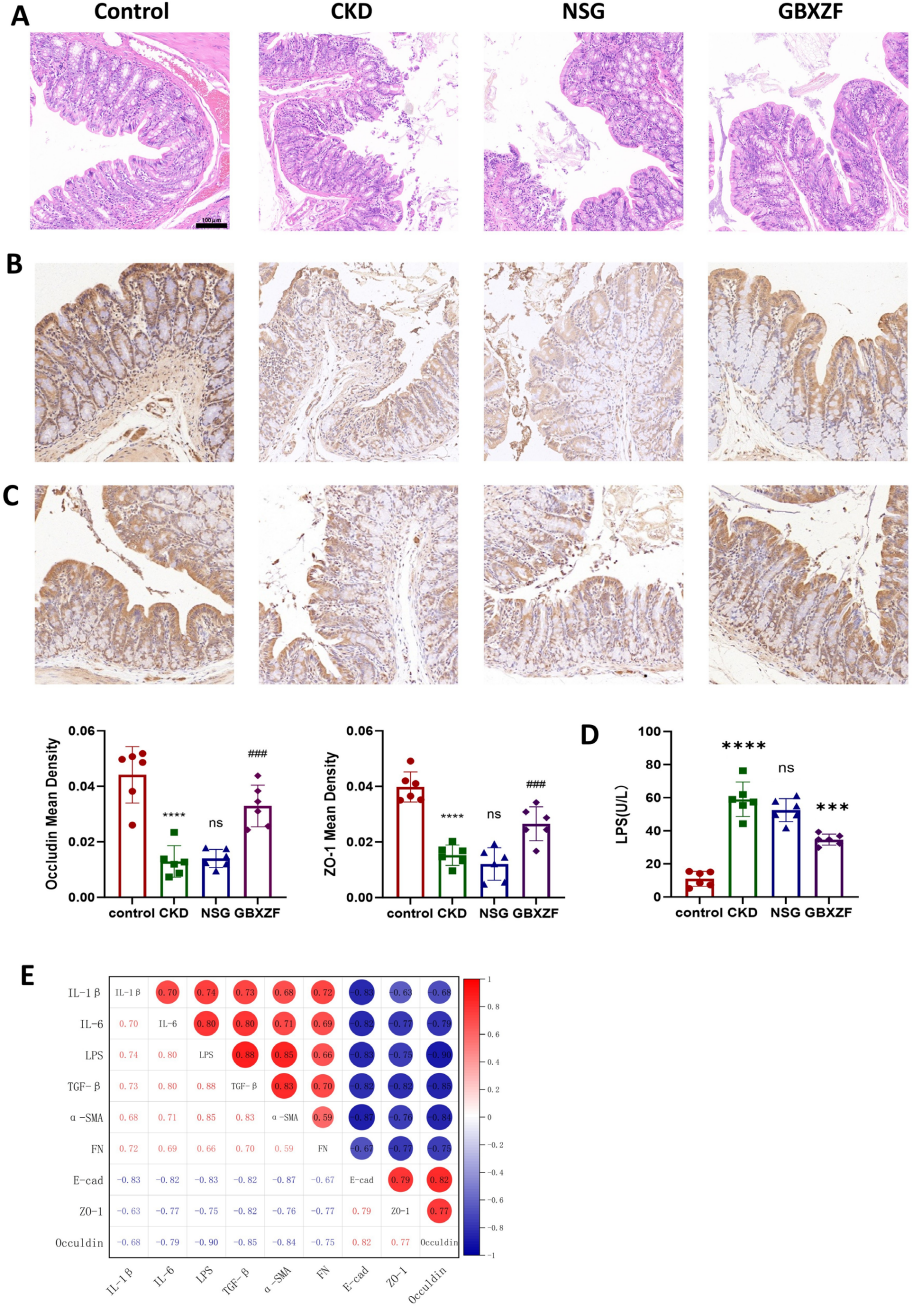
GBXZF repairs intestinal barrier damage. (A) Representative pathological sections of colon stained by H&E. Immunohistochemistry images and protein mean densities of (B) Occludin and (C) ZO‐1 in the colon tissues of the rats from the control, CKD, and GBXZF groups. (D) LPS levels in blood. (E) Correlation diagram. Scale bar = 100 μm; magnification, × 20. Data represent mean ± SD (*n* = 6). *p < 0.05, **p < 0.01,***p < 0.001 and ****p < 0.0001.

The mechanism of improvement of renal fibrosis by traditional Chinese medicine is often related to improving intestinal flora disorders and repairing the intestinal barrier. Therefore, we also performed pathological and immunohistochemical analyses on the colon of four groups of rats. Compared with the CONTROL group, the CKD and NSG groups exhibited severe mucosal damage (epithelial erosion, crypt loss, and inflammatory infiltration) in HE staining (Figure 5A) and significantly downregulated expression of tight junction proteins ZO‐1 and Occludin (Figure 5B,C). In contrast, GBXZF treatment markedly improved mucosal architecture and significantly upregulated ZO‐1/Occludin expression vs. NSG. Critically, GBXZF administration significantly reduced serum LPS levels compared to NSG, contributing to lowered systemic inflammation (Figure 5D). Figure 5E shows the correlation between blood inflammatory factors, indicators of colonic damage, and indicators of renal fibrosis. Our results show that inflammatory factors in blood are significantly negatively correlated with colonic damage indicators and positively correlated with renal fibrosis indicators.

### 3.6 GBXZF Upregulated PI3K/AKT Pathway

KEGG enrichment analysis and molecular docking suggested the involvement of the AGE‐RAGE pathway and emphasized the potential importance of the PI3K/AKT pathway in the mechanism of action of GBXZF in ameliorating renal fibrosis; thus, we validated the key targets and downstream factors of this pathway.

**FIGURE 6:**
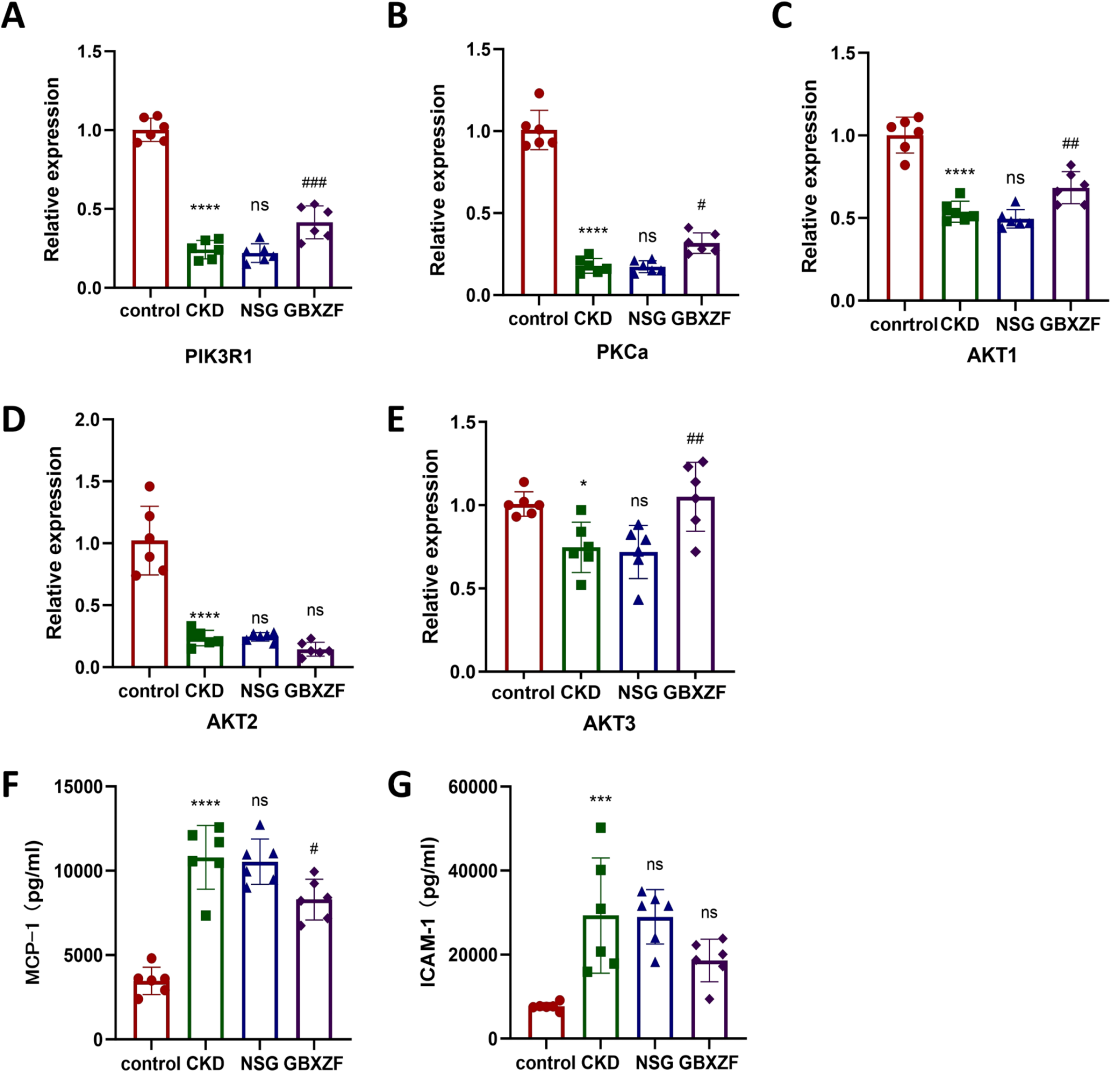
GBXZF upregulated PI3K/AKT pathway. Relative expression of (A) PIK3R1, (B) PKCα, (C) AKT1, (D) AKT2, and (E) AKT3 in kidney tissues of four groups of rats. The serum levels of (F) MCP‐1, and (G) ICAM‐1. Data represent mean ± SD (*n* = 6). *p < 0.05, **p < 0.01,***p < 0.001 and ****p < 0.0001.

We used qpcr and Elisa to detect the key targets and downstream inflammatory factors in the PI3K/AKT pathway. Compared with the control group, the expression of key targets of the PI3K/AKT pathway PIK3R1, PKCa, AKT1, AKT2, and AKT3 was significantly decreased in the CKD group. There was no significant change in the indices of both groups in the NSG and CKD groups. In contrast, PIK3R1, PKCa, AKT1, and AKT3 targets were significantly upregulated in the GBXZF group compared with the NSG group, and AKT2 targets also showed a trend of upregulation, indicating that GBXZF could upregulate the expression of PI3K/AKT pathway‐related genes (Figures 6A–E). Elisa results showed that the serum levels of two inflammatory factors, MCP‐1 and ICAM‐1, were significantly elevated in CKD rats compared with the control group. Compared with the CKD group, there was no significant change in the serum levels of inflammatory factors in rats in the NSG group. In contrast, the inflammatory factors in the serum of rats in the GBXZF group were significantly reduced compared with those in the NSG group, suggesting that GBXZF could ameliorate renal fibrosis by upregulating the PI3K/AKT inflammatory pathway, which was associated with a reduction in the levels of inflammatory factors in the blood (Figure 6F,G).

## 4 Discussion

**FIGURE 7:**
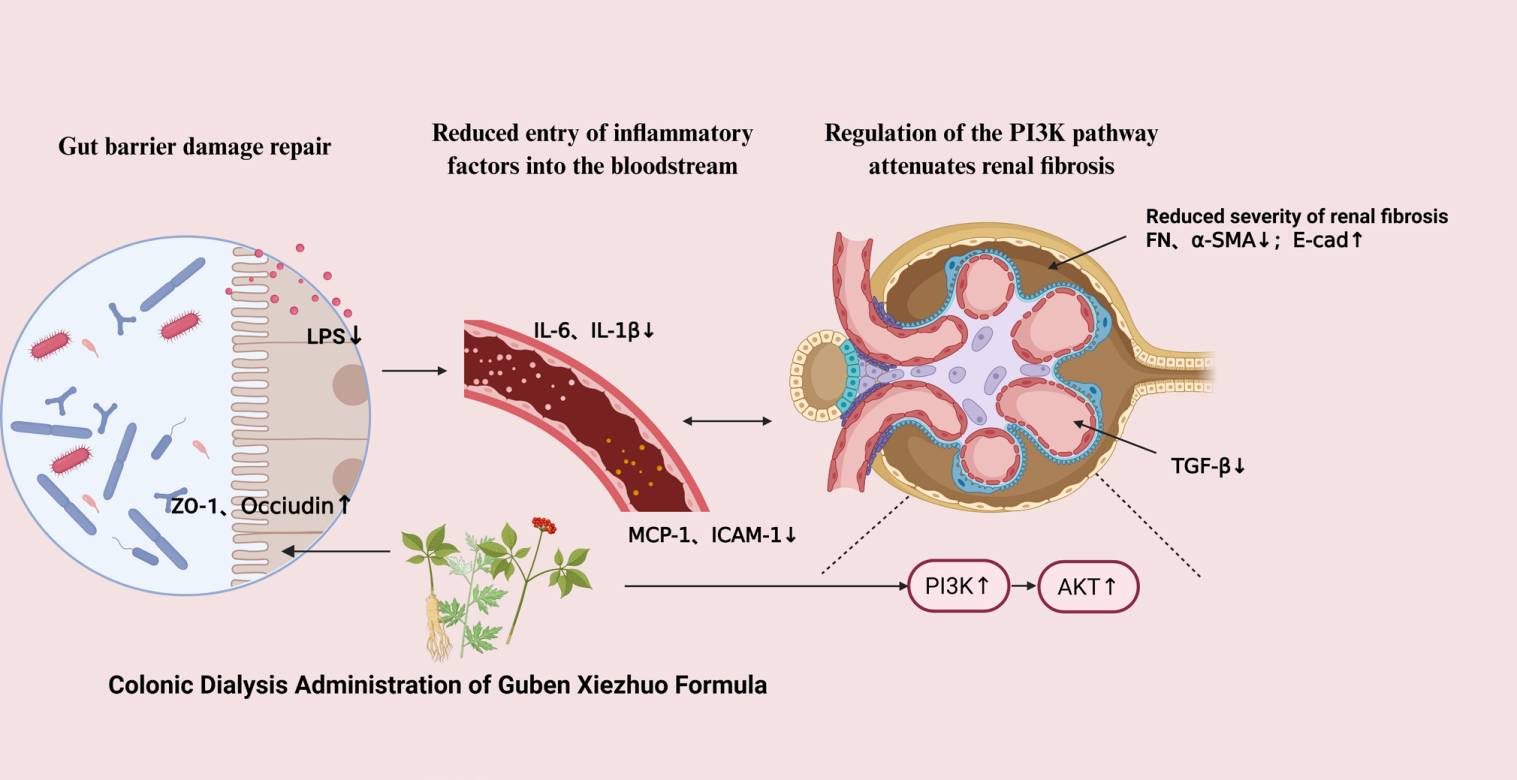
Mechanism of action of GBXZF in ameliorating renal fibrosis. Schematic diagram illustrating that GBXZF, administered by colonic dialysis, repairs the intestinal barrier to reduce the systemic influx of LPS and pro‐inflammatory factors. This subsequently leads to the upregulation of the PI3K/AKT signaling pathway, ultimately attenuating renal fibrosis.

Accumulating evidence indicates that Chinese herbal medicines possess ameliorative effects on renal fibrosis, with their mechanisms of action becoming increasingly elucidated. These herbal interventions can mitigate renal fibrosis and delay the progression of renal diseases through multiple pathways, including modulation of gut microbiota, repair of the intestinal barrier, participation in metabolic reactions, inhibition of inflammatory pathways, reduction of oxidative stress, and attenuation of cellular apoptosis and injury. In the present study, we employed network pharmacology and molecular docking techniques to predict the specific mechanisms by which GBXZF ameliorates renal fibrosis. Using a 0.5% adenine‐induced renal fibrosis model in rats, we demonstrated that GBXZF colonic dialysis improves renal fibrosis by repairing the intestinal barrier and reducing inflammatory factors in the blood, thereby intervening in the PI3K/AKT signaling pathway. This study highlights the therapeutic advantages of traditional Chinese medicine in treating renal fibrosis through multi‐target interventions, with the specific mechanisms illustrated in Figure 7.

This formula uses cooked epiphyllum as the ruler herb to warm Yang and drain dampness, addressing spleen‐kidney Yang deficiency in late‐stage renal failure. Red ginseng and rhubarb serve as ministers to replenish qi and remove turbidity, while dandelion and safflower act as adjutants to clear heat and activate blood. Oyster shell guides the formula downward as the envoy. The combination works to delay renal failure progression and regulate immune function. Guided by the TCM concept of “homology of medicine and food”, this formula intentionally selects ingredients that are not only therapeutically effective but also nutritionally supportive. In this formula, red ginseng, dandelion, and oyster shell are all common ingredients in our daily diet. They simultaneously address the fundamental deficiency and superficial heat symptoms characteristic of chronic kidney disease. Red ginseng, dandelion, and oyster are all classic examples of an ingredient belonging to the food‐medicine continuum, exhibiting synergistic effects in the treatment of CKD. Red ginseng functions to warm the kidney and strengthen Yang, as well as fortify the spleen and boost qi. Its bioactive components, including saponins and polysaccharides, exhibit antioxidant and anti‐inflammatory activities, and contribute to the amelioration of anemia, thereby potentially retarding the progression of CKD (Fang et al. 2023; Liu et al. 2020; Mohanan et al. 2018). Dandelion possesses properties to clear heat, promote diuresis, detoxify, and disperse nodules. The flavonoids abundant in dandelion have been shown to counteract renal interstitial fibrosis and regulate water metabolism (Au et al. 2025; Fan et al. 2023). Oyster can subdue yang, soften hardness, nourish yin, and calm the spirit. Its active constituents, such as taurine and zinc, inhibit renal inflammation and fibrosis (Wang et al. 2021; Zhang et al. 2023). In combination, these constituents synergistically integrate nutritional support with therapeutic intervention, collectively modulating the characteristic pathogenic conditions of “turbid toxin” and “damp‐heat” in chronic kidney disease.

Network pharmacology technology can establish the relationship between the active ingredients, targets, and disease, consistent with the holistic view of Chinese medicine (Shi et al. 2014). The network pharmacology and molecular docking results showed that the GBXZF multicomponent could improve renal fibrosis by modulating multiple targets through relevant metabolic pathways. Among them, the active component of hydroxy saffron yellow pigment A isomer has the most significant degree of value in the network. Hydroxy saffron yellow pigment A can inhibit the expression of NLRP3 inflammatory vesicle‐associated proteins through downregulation of LPS, etc., inhibit the activation of NLRP3 inflammatory vesicles, and attenuate renal fibrosis (Li et al. 2019; Xu et al. 2016). In molecular docking assays, the hydroxy saffron yellow pigment A isomer also showed vigorous binding activity with AKT1 and JUN, indicating that it can intervene in the inflammatory pathway and has good potential to reduce the inflammatory response and thus ameliorate renal fibrosis. The PPI network analysis showed that the targets of AKT1, JUN, TNF, HSP90AA1, and IL‐6 are the GBXZF action of GBXZF. The molecular docking results also proved that these targets have good binding ability with GBXZF active ingredients and are potential bioactive targets. These targets are mainly enriched in the AGE‐RAGE signaling pathway, especially the PI3K/AKT signaling pathway, so we paid special attention to this inflammatory pathway and used animal experiments to verify it.

We first assessed the extent of renal fibrosis by evaluating inflammatory factors or proteins associated with renal fibrosis, such as TGF‐β, FN, α‐SMA, and E‐cad. TGF‐β is an inflammatory factor that is closely associated with fibrosis and acts through the activation of downstream factors such as Smad2 and Smad3 or other atypical signaling factors, inducing the proliferation of myofibroblasts and leading to their aggregation in the extracellular matrix, which in turn leads to the development of a wide range of fibrotic diseases (Frangogiannis 2020; Peng et al. 2022; Song et al. 2022). Kayhan et al. (2023) demonstrated that TGF‐β is essential in reducing the inflammatory response, ameliorating renal fibrosis, and improving renal function. The network pharmacology and molecular docking results showed that TGF had a good binding capacity with many active ingredients in GBXZF. The results of animal experiments showed that, compared with the NSG group, the expression of TGF‐β was significantly reduced in the kidney tissues of rats in the GBXZF group, and proteins such as FN and α‐SMA, which can show excessive deposition of ECM, were also significantly reduced, which indicated that GBXZF could reduce the deposition of ECM and improve the fibrosis of the kidney. Further research is needed to clarify the precise molecular targets and optimize therapeutic strategies for fibrosis treatment.

Improvement in renal function has been reported to be associated with improvement in intestinal microecology (Wang, Feng, et al. 2022; Xie et al. 2022). Our previous study found that CKD patients have dysbiosis and metabolic disorders and that *Faecalibacterium prausnitzii* ( *F. prausnitzii* ) is significantly reduced compared to healthy individuals. Supplementation with *F. prausnitzii* in CKD mice increases short‐chain fatty acids (SCFAs), reduces serum levels of various uremic toxins, and improves renal microinflammation and renal fibrosis via G‐protein‐coupled receptor 43 (GPR43) (Li, Xu, et al. 2022). We speculated that the therapeutic effect of GBXZF on renal fibrosis might be related to improving intestinal microecology. Therefore, we assessed colonic injury by histology and assays for key injury markers. The results of animal experiments showed that compared with the NSG group, rats in the GBXZF group showed significantly reduced intestinal injury, significantly reduced epithelial cell shedding in the mucosal layer, significantly increased levels of intestinal tight junction proteins ZO‐1 and Occludin, and a repaired intestinal barrier. These results can prove that GBXZF has a certain repairing effect on intestinal barrier damage. In turn, repair of intestinal barrier damage leads to a reduction in the entry of endotoxins such as LPS into the bloodstream and a significant reduction in the levels of two inflammatory factors, IL‐1β and IL‐6. We performed a correlation analysis of these indicators, and the results showed that LPS, IL‐1β, and IL‐6 were significantly negatively correlated with the intestinal barrier indicators ZO‐1 and Occludin, which suggests that the repair of intestinal barrier damage by GBXZF suppresses systemic inflammatory responses.

Although GBXZF's regulation of the gut‐kidney axis provides an explanation for its therapeutic efficacy, the intracellular signaling events downstream remain poorly characterized. Therefore, we employed network pharmacology analysis to screen for key signaling pathways. Molecular docking further validated the stable binding potential between GBXZF's key components and core targets within the PI3K/AKT pathway. This computational evidence collectively posits the PI3K/AKT pathway as a potential core mediator. The PI3K/AKT signaling pathway is a critical intracellular signaling network, and PI3K and AKT are the key proteins of the pathway. PI3K is an intracellular phosphatidylinositol kinase, which is involved in a variety of cellular physiological processes such as cell proliferation, metabolism, and survival, etc., which can activate AKT, and activated AKT can play corresponding biological roles by phosphorylating, activating, or inhibiting a series of downstream substrates (Franke et al. 2003; Wang et al. 2023). AKT activates or inhibits downstream substrates through phosphorylation, thus playing corresponding biological roles. It has been shown that intervening in this inflammatory pathway can reduce the inflammatory response by inhibiting the NF‐κB pathway, among other things, thereby improving the organism's state (Alsereidi et al. 2024; Li et al. 2018). Wang et al. (Wang et al. 2019) found that upregulation of the PI3K/AKT signaling pathway activates the GSK‐3 β/Nrf2 pathway and inhibits the ASK1/JNK signaling pathway, which in turn reduces oxidative stress, inhibits inflammatory responses, and improves kidney function. Yang et al. (2022) also demonstrated that PrPC can improve CKD by upregulating PI3K/Akt/mTOR signaling and cell proliferation. Combined with the results of our network pharmacology, that is, both AKT1 and IL‐6 have a good binding ability with various active ingredients of GBXZF, we speculated that GBXZF could reduce the inflammatory response and improve renal fibrosis by upregulating the PI3K/AKT signaling pathway. Therefore, we conducted animal experiments to verify it. Our qPCR analysis revealed significant alterations in the gene expression of key components of the PI3K/AKT pathway, suggesting its potential involvement in the therapeutic mechanism. However, it is important to note that these findings at the transcriptional level do not fully substantiate pathway activation, as mRNA levels may not always correlate with protein activity and phosphorylation status (Vogel and Marcotte 2012). Therefore, while our data point to a role for the PI3K/AKT pathway, further validation at the protein level is warranted to conclusively establish its contribution.

## 5 Conclusion

This study demonstrates that GBXZF administered via colonic dialysis can ameliorate renal fibrosis by repairing the intestinal barrier, reducing LPS and inflammatory factor entry into the bloodstream, and upregulating the PI3K/AKT pathway. These findings highlight the multi‐targeted effects of GBXZF on the gut‐kidney axis and provide mechanistic evidence for its anti‐fibrotic effects. Notably, GBXZF features food‐borne ingredients like Red Ginseng, Dandelion, and Oyster, supporting the use of colonic dialysis as an effective route of administration of herbal medicine for the treatment of renal fibrosis, providing a new therapeutic strategy for the treatment of chronic kidney disease.

## Author Contributions

Gao Zitian, Tang Yuyan, He Haidong, and Chen Yu designed the research and interpreted the data. Huang Luyan, Xu Jiahui, and Sun Weiqian performed network pharmacology and molecular docking analysis. Gao Zitian, Huang Luyan, Tang Yuyan, and Huang Luyan conducted animal experiments. Gao Zitian, He Haidong, and Chen Yu wrote the manuscript. He Haidong, and Chen Yu reviewed the article. All authors have read and agreed to the published version of the manuscript.

## Disclosure

The authors have nothing to report.

## Ethics Statement

Animal experiments were approved by the Animal Ethics Committee of the Minhang Hospital, Fudan University (Shanghai, China; *No.2023‐MHFY‐26JZS*).

## Supporting information


**Figure S1:** Spectra of identification results of GBXZF. (A) UPLC‐HRMS Base Peak Ion Flow Chart (BPC)‐Negative Ion Mode of GBXZF. (B) UPLC‐HRMS Base Peak Ion Flow Chart (BPC)—Positive Ion Mode of GBXZF. (C) Ultraviolet Chromatogram of GBXZF—UV 254 nm.

**Table S1:** Composition of Guben Xiezhuo Formula.

## Data Availability

The data that supports the findings of this study are available in the Supporting Information of this article.
