# Supplementary figures and images for: A New Strategy for Treating Renal Fibrosis Based on a Drug‐Food Homogeneous Formula of Traditional Chinese Medicine
Source: Food Sci Nutr. 2025 Nov 14;13(11):e71186. doi: 10.1002/fsn3.71186 (PMC12616506; doi:10.1002/fsn3.71186)

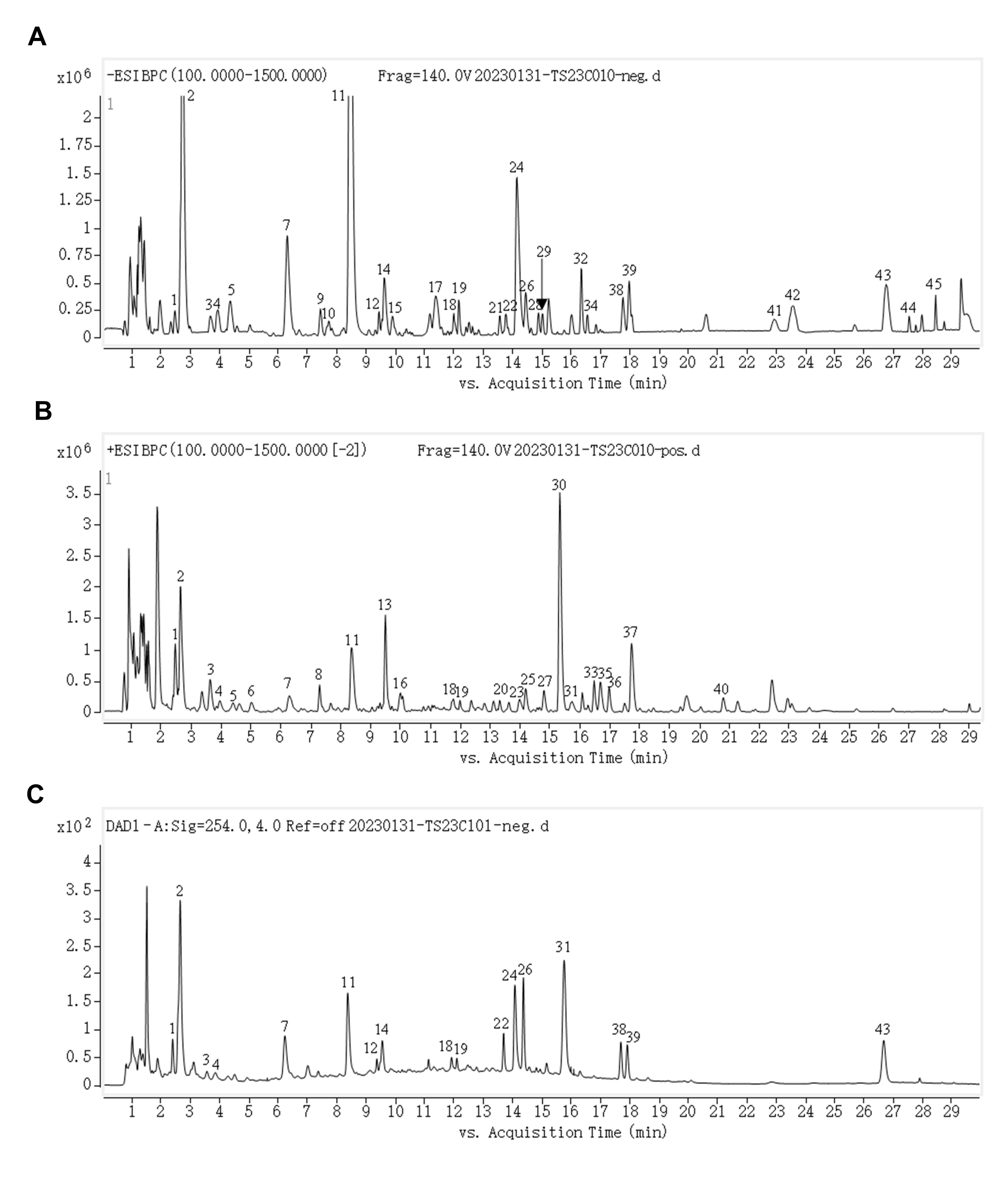

Supplement: Supplementary file 1 — Figure S1: Spectra of identification results of GBXZF. (A) UPLC‐HRMS Base Peak Ion Flow Chart (BPC)‐Negative Ion Mode of GBXZF. (B) UPLC‐HRMS Base Peak Ion Flow Chart (BPC)—Positive Ion Mode of GBXZF. (C) Ultraviolet Chromatogram of GBXZF—UV 254 nm. [file FSN3-13-e71186-s001.jpg]
