# Supplementary material for: A New Strategy for Treating Renal Fibrosis Based on a Drug‐Food Homogeneous Formula of Traditional Chinese Medicine
Source: Food Sci Nutr. 2025 Nov 14;13(11):e71186. doi: 10.1002/fsn3.71186 (PMC12616506; doi:10.1002/fsn3.71186)
Supplement: Supplementary file 2 — Table S1: Composition of Guben Xiezhuo Formula. [file FSN3-13-e71186-s002.docx]

| Serial number | time（min） | adduct ion | *m/z actual value* | *m/z theoretical value* | molecular formula | molecular mass | name | origin |
| --- | --- | --- | --- | --- | --- | --- | --- | --- |
| 1 | 2.47 | [M-H]^-^ | 282.0850 | 282.0839 | C_10_H_13_N_5_O_5_ | 283.09 | Guanosine | All |
| 2 | 2.67 | [M-H]^-^ | 331.0675 | 331.0665 | C_13_H_16_O_10_ | 332.07 | 1-O-Galloyl-β-D-glucose | Prepared Rhubarb |
| 3 | 3.68 | [M-H]^-^ | 493.1206 | 493.1194 | C_19_H_26_O_15_ | 494.13 | 1'-O-Galloylsucrose | Prepared Rhubarb |
| 4 | 3.93 | [M-H]^-^ | 611.1625 | 611.1612 | C_27_H_32_O_16_ | 612.17 | Hydroxysafflor yellow A Isomer | Carthamus Flower |
| 5 | 4.36 | [M-H]^-^ | 611.1636 | 611.1612 | C_27_H_32_O_16_ | 612.17 | Hydroxysafflor yellow A Isomer | Carthamus Flower |
| 6 | 5.02 | [M+H]^+^ | 217.0976 | 217.0977 | C_12_H_12_N_2_O_2_ | 216.09 | 1,2,3,4-Tetrahydro-β-carboline-3-carboxylic acid | Dandelion |
| 7 | 6.30 | [M-H]^-^ | 311.0415 | 311.0403 | C_13_H_12_O_9_ | 312.05 | Caftaric acid | Dandelion |
| 8 | 7.30 | [M+H]^+^ | 486.2707 | 486.2703 | C_24_H_39_NO_9_ | 485.26 | Mesaconine | Prepared Aconite |
| 9 | 7.44 | [M-H]^-^ | 421.1152 | 421.1135 | C_20_H_22_O_10_ | 422.12 | / | / |
| 10 | 7.73 | [M-H]^-^ | 239.0558 | 239.0556 | C_11_H_12_O_6_ | 240.06 | Eucomic acid | Prepared Aconite |
| 11 | 8.46 | [M-H]^-^ | 611.1652 | 611.1612 | C_27_H_32_O_16_ | 612.17 | Hydroxysafflor yellow A | Carthamus Flower |
| 12 | 9.43 | [M-H]^-^ | 801.1755 | 801.1726 | C_33_H_38_O_23_ | 802.18 | 6-hydroxykaempferol 3,6-di-O-β-D-glucoside-7-O-β-D-glucuronide | Carthamus Flower |
| 13 | 9.50 | [M+H]^+^ | 454.2809 | 454.2805 | C_24_H_39_NO_7_ | 453.27 | Fuziline | Prepared Aconite |
| 14 | 9.62 | [M-H]^-^ | 353.0877 | 353.0873 | C_16_H_18_O_9_ | 354.10 | Chlorogenic acid | Dandelion |
| 15 | 9.91 | [M-H]^-^ | 177.0191 | 177.0188 | C_9_H_6_O_4_ | 178.03 | Esculetin | Dandelion |
| 16 | 10.08 | [M+H]^+^ | 438.2867 | 438.2855 | C_24_H_39_NO_6_ | 437.28 | Neoline | Prepared Aconite |
| 17 | 11.37 | [M-H]^-^ | 611.1631 | 611.1612 | C_27_H_32_O_16_ | 612.17 | Hydroxysafflor yellow B | Carthamus Flower |
| 18 | 12.00 | [M-H]^-^ | 771.2011 | 771.1984 | C_33_H_40_O_21_ | 772.21 | 6-Hydroxykaempferol 3-rutinoside -6-glucoside | Carthamus Flower |
| 19 | 12.16 | [M-H]^-^ | 625.1426 | 625.1405 | C_27_H_30_O_17_ | 626.15 | 6-Hydroxykaempferol 3,6-diglucoside | Carthamus Flower |
| 20 | 13.31 | [M+H]^+^ | 606.2916 | 606.2914 | C_31_H_43_NO_11_ | 605.28 | 14-Benzoyl-10-hydroxymesaconine | Prepared Aconite |
| 21 | 13.55 | [M-H]^-^ | 477.1403 | 477.1397 | C_23_H_26_O_11_ | 478.15 | Lindleyin | Prepared Rhubarb |
| 22 | 13.77 | [M-H]^-^ | 431.0985 | 431.0978 | C_21_H_20_O_10_ | 432.11 | Aloe-emodin-8-O-β-D-glucopyranoside | Prepared Rhubarb |
| 23 | 13.99 | [M+H]^+^ | 1045.2818 | 1045.2825 | C_48_H_52_O_26_ | 1044.27 | Anhydrosafflor yellow B | Carthamus Flower |
| 24 | 14.15 | [M-H]^-^ | 473.0732 | 473.0720 | C_22_H_18_O_12_ | 474.08 | Chicoric acid | Dandelion |
| 25 | 14.18 | [M+H]^+^ | 233.0817 | 233.0814 | C_13_H_12_O_4_ | 232.07 | 2-Methyl-5-acetonyl-7-hydroxychromone | Prepared Rhubarb |
| 26 | 14.45 | [M-H]^-^ | 445.0790 | 445.0771 | C_21_H_18_O_11_ | 446.08 | Rhein-8-O-β-D-glucoside | Prepared Rhubarb |
| 27 | 14.80 | [M+H]^+^ | 235.0977 | 235.0970 | C_13_H_14_O_4_ | 234.09 | 7-Hydroxy-2-(2-hydroxypropyl)-5-methyl-4H-1-benzopyran-4-one | Prepared Rhubarb |
| 28 | 14.88 | [M-H]^-^ | 593.1527 | 593.1507 | C_27_H_30_O_15_ | 594.16 | Kaempferol-3-O-rutinoside | Carthamus Flower |
| 29 | 15.03 | [M-H]^-^ | 623.1629 | 623.1612 | C_28_H_32_O_16_ | 624.17 | Narcissoside | Carthamus Flower |
| 30 | 15.33 | [M+H]^+^ | 590.2988 | 590.2965 | C_31_H_43_NO_10_ | 589.29 | Benzoylmesaconine | Prepared Aconite |
| 31 | 15.74 | [M+H]^+^ | 149.0594 | 149.0602 | C_9_H_8_O_2_ | 148.05 | Cinnamic acid | Prepared Rhubarb、Prepared Aconite |
| 32 | 16.34 | [M+FA-H]^-^ | 845.4933 | 845.4898 | C_42_H_72_O_14_ | 800.49 | Ginsenoside Rg1 | Red Ginseng |
| 33 | 16.48 | [M+H]^+^ | 604.3135 | 604.3121 | C_32_H_45_NO_10_ | 603.30 | Benzoylaconitine | Prepared Aconite |
| 34 | 16.55 | [M+FA-H]^-^ | 991.5527 | 991.5477 | C_48_H_82_O_18_ | 946.55 | Ginsenoside Re | Red Ginseng |
| 35 | 16.70 | [M+H]^+^ | 191.0714 | 191.0708 | C_11_H_10_O_3_ | 190.06 | 2,5-Dimethyl-7-hydroxychromone | Prepared Rhubarb |
| 36 | 16.97 | [M+H]^+^ | 574.3032 | 574.3016 | C_31_H_43_NO_9_ | 573.29 | Benzoylhypacoitine | Prepared Aconite |
| 37 | 17.72 | [M+H]^+^ | 679.5134 | 679.5122 | C_36_H_66_N_6_O_6_ | 678.50 | Cyclic hexaleucine | / |
| 38 | 17.77 | [M-H]^-^ | 431.0992 | 431.0978 | C_21_H_20_O_10_ | 432.11 | Aloe-emodin-3-(hydroxymethyl)-O-β-D-glucopyranoside | Prepared Rhubarb |
| 39 | 17.98 | [M-H]^-^ | 431.0996 | 431.0978 | C_21_H_20_O_10_ | 432.11 | Emodin-8-O-glucoside | Prepared Rhubarb |
| 40 | 20.80 | [M+H]^+^ | 616.3135 | 616.3121 | C_33_H_45_NO_10_ | 615.30 | Hypaconitine | Prepared Aconite |
| 41 | 22.93 | [M-H]^-^ | 297.0402 | 297.0399 | C_16_H_10_O_6_ | 298.05 | 6-Methyl-rhein | Prepared Rhubarb |
| 42 | 23.55 | [M-H]^-^ | 329.2338 | 329.2328 | C_18_H_34_O_5_ | 330.24 | 9,12,13-Trihydroxy-10-octadecenoic acid | All |
| 43 | 26.76 | [M-H]^-^ | 283.0251 | 283.0243 | C_15_H_8_O_6_ | 284.03 | Rhein | Prepared Rhubarb |
| 44 | 27.54 | [M-H]^-^ | 1107.6008 | 1107.5951 | C_54_H_92_O_23_ | 1108.60 | Ginsenoside Rb1 | Red Ginseng |
| 45 | 28.45 | [M-H]^-^ | 311.2228 | 311.2223 | C_18_H_32_O_4_ | 312.23 | 12,13-dihydroxy-9Z,15Z-octadecadienoic acid | All |
